# Risk factors of intracranial infection in patients after intracranial aneurysm surgery: Implication for treatment strategies

**DOI:** 10.1097/MD.0000000000027946

**Published:** 2021-12-03

**Authors:** Xiaohong Guo, Junkang Fang, Yi Wu

**Affiliations:** Department of Neurosurgery, Dongyang People Hospital, Jinhua, China.

**Keywords:** aneurysm, care, intracranial infection, surgery, treatment

## Abstract

Postoperative intracranial infection after intracranial aneurysm is relatively common in clinical setting; it is necessary to analyze the clinical risk factors of postoperative intracranial infection, to provide reliable evidence to the management of aneurysm.

Patients with intracranial aneurysm admitted from January 1, 2016, to November 30, 2020, are included. We collected the patient's personal and treatment data, and analyzed the risk factors of intracranial infection by multivariate logistic regression analysis. We compared the cerebrospinal fluid (CSF) indicators and serological indicators and analyzed their correlation with intracranial infection by spearman analysis.

A total of 236 patients with intracranial aneurysm were included; the incidence of postoperative intracranial infection was 12.71%. There were significant differences in the diabetes, intraoperative aneurysm rupture, intraoperative CSF leakage, duration of surgery, and estimated blood loss between infection and non-infection group. Logistic regression indicated that diabetes [odds ratio (OR) 2.053, 95% confidence interval (95% CI) 1.092∼3.385], intraoperative aneurysm rupture (OR 2.239, 95% CI 1.173∼4.312), intraoperative CSF leakage (OR 2.168, 95% CI 1.033∼3.451), duration of surgery ≥360 minutes (OR 1.926, 95% CI 1.108∼2.655), and estimated blood loss ≥125 mL (OR 2.459, 95% CI 1.854∼3.447) were the independent risk factors of postoperative intracranial infection in patients with aneurysm surgery (all *P* < .05). *Klebsiella pneumoniae*, *Escherichia coli*, and *Staphylococcus epidermidis* were the top 3 commonly seen pathogens. Spearman analyses indicated that PCT, CRP, LA, LDH were all correlated with intracranial infection (all *P* < .05).

There are multiple factors for the postoperative intracranial infection in patients with aneurysm. Coping strategies should be formulated targeted on those risks to improve the prognosis of patients.

## 1 Introduction

Intracranial aneurysms are the main cause of subarachnoid hemorrhage (SAH), accounting for about 70% of SAH.^[1]^ In all cerebrovascular accidents, aneurysm rupture and hemorrhage is second only to cerebral thrombosis and hypertensive cerebral hemorrhage, and the mortality is very high.^[2]^ At present, craniotomy aneurysm clipping is still the first choice for surgical treatment. The current mortality rate of microsurgery has dropped to less than 2%.^[3]^ However, due to the difficulty of the operation and the long operation time, the incidence of intracranial infection after craniotomy for intracranial aneurysms ranges from 2.6% to 30.0%, and the mortality is over 30.0%.^[4,5]^ Therefore, the early control of intracranial infection and the improvement of patient prognosis are of positive significance, and the early indicators of intracranial infection should be comprehensively analyzed in clinical treatment.

Intracranial infection is a common complication after neurosurgical aneurysm surgery, and it is an important cause of prolonged unhealing, even disability, and death.^[6]^ The reason for the infection is that on the one hand, the original disease changes the intracranial environment and the patient's own physical condition.^[7]^ On the other hand, the craniotomy also destroys the original tissue integrity, creating the possibility of bacterial invasion.^[8]^ The occurrence of intracranial infection not only caused the patient's long-term treatment, but also affected the patient's prognosis, and caused tremendous pressure on the family and society.^[9,10]^ Therefore, medical staff in neurosurgery and related departments should pay enough attention to the control of postoperative infection. Therefore, the purpose of this study is to explore the relevant clinical influencing factors of intracranial infection after intracranial aneurysm surgery, and to provide a scientific basis for the clinical treatment of intracranial aneurysms.

## 2 Methods

### 2.1 Ethics

In this study, all methods were performed in accordance with the relevant guidelines and regulations. Our study had been verified and approved by the ethical committee of our hospital (No.1605148), and written informed consents had been obtained from all the included patients.

### 2.2 Patients

We selected patients with intracranial aneurysm admitted from January 1, 2016, to November 30, 2020, as the study population. The inclusion criteria of the patients were as follows:

(1) All patients met the diagnostic criteria for aneurysms based on the “Expert Consensus on the Diagnosis and Treatment of Neurosurgery Aneurysms in China";^[11,12]^
(2) Age> 20 years;
(3) Postoperative survival time> 1 week;
(4) Patients were well informed and agreed to participate in this study.

The exclusion criteria for patients were patients with intracranial infections such as meningitis and brain abscess; patients with intracranial vascular malformations and cerebral hemorrhage; patients with immune dysfunction; and patients who were unwilling to participate in the study.

### 2.3 Treatments

Before surgery, all patients were given Bayaspirin 80 mg and Clopidogrel 60 mg once daily for 3 consecutive days. All patients underwent intracranial aneurysm surgery under general anesthesia, and appropriate stents were selected according to the patient's condition. The surgery was performed by the same group of doctors. During the operation and 4 hours after the operation, ceftriaxone 2 g was routinely administered intravenously, once a day. If intracranial infection was confirmed postoperatively, oral ceftriaxone treatment was continuously given for 1 month.

### 2.4 Diagnostic criteria of intracranial infection

The diagnostic criteria for intracranial infection^[12]^ in this study were: ① The patient's body temperature exceeded 38°C or below 36°C; ② Clinical symptoms had clear meningeal irritation, symptoms of increased intracranial pressure or clinical imaging evidence; ③ White blood cells> 10∗10^9^ /L or the proportion of neutrophils>80%; ④ Cerebrospinal fluid (CSF) analysis showed that the total number of white blood cells >500∗10^6^/L, multinucleated cells >80%, blood sugar <4.5 mmol/L. A positive bacterial smear or a positive cerebrospinal fluid bacteriological culture was necessary to confirm the diagnosis of intracranial infection.

### 2.5 Data collection

The 2 authors collected the gender, age, body mass index (BMI), hypertension, diabetes, hyperlipidemia, intraoperative aneurysm rupture, CSF leakage, intraoperative bleeding, duration of surgery, and other clinical data of all included patients.

In this study, intraoperative CSF leakage was defined as the communication between the CSF cavity and the outside of the skull. It was judged by the presence of clear liquid or light red bloody fluid in the surgical incision. When the drainage volume reached 30 mL (before the intracranial infection was confirmed) in all patients, 3 mL of CSF specimens were collected and submitted for testing following indicators: procalcitonin (PCT), C-reactive protein (CRP), lactic acid (LA), lactic dehydrogenase (LDH), and we analyzed and collected the bacterial composition of postoperative CSF culture.

Meanwhile, 5 mL of peripheral venous blood was drawn from all patients on an empty stomach the next morning after surgery, centrifuged with a centrifugal radius of 15 cm and 3000 r/min for 10 minutes, and the supernatant was taken and stored at -80°C. Aspartate transaminase (AST), LDH, LDH-1 levels were detected by ELISA method, and the detection reagents were purchased from Shanghai JiaHua Enzyme-linked Reagent Co., Ltd (Shanghai, China). The detection process was carried out in strict accordance with the instructions. The serum levels of LDH, LDH-1 were compared and analyzed between the 2 groups.

### 2.6 Statistical analysis

We used SPSS24.00 (International Business Machines Corporation (USA)) software for statistical analysis. Count data were expressed as rate (%), comparison between groups was conducted by chi-square test; measurement data was expressed as mean ± standard deviation, and comparison between groups was conducted by independent sample *t* test. A single factor analysis was performed for each factor, and statistically significant factors were further included in the multivariate logistic regression analysis to analyze the risk factors of intracranial infection. Spearman analysis was used to explore the correlation between the detection indicators and the incidence of postoperative intracranial infection. In this study, *P* < .05 was regarded as the difference with significant statistical significance.

## 3 Results

### 3.1 The characteristics of included patients

A total of 236 patients with intracranial aneurysm were included, of whom 30 patients had the intracranial infection, and the incidence of postoperative intracranial infection was 12.71%. As presented in Table 1, there were significant differences in the diabetes, intraoperative aneurysm rupture, intraoperative cerebrospinal fluid leakage, duration of surgery, and estimated blood loss between infection and non-infection groups (all *P* < .05); no significant differences in the gender, age, hypertension, hyperlipidemia, intraoperative blood infusion, and duration of hospital stay were found (*P* > .05).

**Table 1:** The characteristics of included patients.

Variables	Infection group (n = 30)	Non-infection group (n = 206)	*t*/χ^2^	*P*
Male/female	17/13	112/94	1.533	.085
Age, yr	41.98 ± 5.25	42.03 ± 5.19	1.217	.071
BMI, kg/m^2^	24.14 ± 3.92	24.55 ± 4.46	1.145	.158
Diabetes	17 (56.67%)	27 (13.11%)	1.183	.011
Hypertension	16 (53.33%)	102 (49.51%)	1.157	.093
Hyperlipidemia	3 (10.00%)	24 (11.65%)	1.124	.103
Intraoperative aneurysm rupture	23 (76.67%)	19 (9.22%)	1.286	.016
Intraoperative cerebrospinal fluid leakage	19 (63.33%)	13 (6.31%)	1.145	.022
Intraoperative blood infusion	16 (53.33%)	98 (47.57%)	1.204	.115
Duration of surgery, min	407.24 ± 75.01	315.45 ± 64.29	29.161	.019
Estimated blood loss, mL	148 (85, 197)	111 (80,166)	4.121	.021
Duration of hospital stay, days	7.30 ± 1.44	5.99 ± 1.02	1.184	.067

### 3.2 Logistic regression analyses

The variable assignments of multivariate logistic regression are presented in Table 2. As Table 3 presented, diabetes [odds ratio (OR) 2.053, 95% confidence interval (95% CI) 1.092∼3.385], intraoperative aneurysm rupture (OR 2.239, 95% CI 1.173∼4.312), intraoperative cerebrospinal fluid leakage (OR 2.168, 95% CI 1.033∼3.451), duration of surgery ≥360 minutes (OR 1.926, 95% CI 1.108∼2.655), and estimated blood loss ≥125 mL (OR 2.459, 95% CI 1.854∼3.447) were the independent risk factors of postoperative intracranial infection in patients with aneurysm surgery (all *P* < .05).

**Table 2:** The variable assignment of multivariate logistic regression.

Factors	Variables	Assignment
Infection	Y	Yes = 1, no = 2
Diabetes	X_2_	Yes = 1, No = 2
Intraoperative aneurysm rupture	X_3_	Yes = 1, no = 2
Intraoperative cerebrospinal fluid leakage	X_4_	Yes = 1, no = 2
Duration of surgery, min	X_5_	≥360 = 1, <360 = 2
Estimated blood loss, mL	X_6_	≥125 = 1, <125 = 2

**Table 3:** Logistic regression analysis on the risk factors of postoperative intracranial infection in patients with aneurysm surgery.

Variables	β	S̄x	OR	95% CI	*P*
Diabetes	0.803	0.149	2.053	1.092∼3.385	.021
Intraoperative aneurysm rupture	0.625	0.122	2.239	1.173∼4.312	.003
Intraoperative cerebrospinal fluid leakage	0.817	0.271	2.168	1.033∼3.451	.017
Duration of surgery ≥360 min	0.633	0.203	1.926	1.108∼2.655	.024
Estimated blood loss ≥125 mL	0.801	0.155	2.459	1.854∼3.447	.009

### 3.3 Pathogens distribution

As presented in Table 4, *Klebsiella pneumoniae, Escherichia coli*, and *Staphylococcus epidermidis* were the top 3 commonly seen pathogens of postoperative intracranial infection.

**Table 4:** Distribution of pathogens of postoperative intracranial infection (n = 32).

Pathogens	Number	Proportion (%)
*Klebsiella pneumoniae*	15	46.88
*Escherichia coli*	6	18.75
*Staphylococcus epidermidis*	4	12.50
*Acinetobacter baumannii*	3	9.38
*Enterococcus faecalis*	2	6.25
*Staphylococcus aureus*	1	3.12
*Pseudomonas aeruginosa*	1	3.12

### 3.4 CSF and serological test results

As indicated in Table 5, there were significant differences in the PCT, CRP, LA, LDH, and AST between infection and non-infection group (all *P* < .05).

**Table 5:** The comparison of cerebrospinal fluid and serological test results.

Variables	Infection group (n = 30)	Non-infection group (n = 206)	*t*/χ^2^	*P*
Cerebrospinal fluid test				
PCT, μg/L	6.16 ± 1.34	2.76 ± 1.29	1.247	.037
CRP, mg/L	24.38 ± 9.85	7.61 ± 2.57	4.288	.040
LA, nmol/L	4.85 ± 1.92	2.84 ± 0.76	1.953	.035
LDH, U/L	309.47 ± 55.33	89.85 ± 32.03	38.174	.001
Serological indicators				
AST, U/L	48.47 ± 21.28	32.11 ± 19.25	14.405	.047
LDH, U/L	304.15 ± 84.36	239.14 ± 61.03	38.172	.001
LDH-1, IU/L	37.34 ± 11.20	30.27 ± 9.11	9.884	.032

As presented in Table 6, Spearman analyses indicated that PCT, CRP, LA, LDH, and AST were all correlated with intracranial infection (all *P* < .05).

**Table 6:** Correlation analysis of patients’ cerebrospinal fluid indexes, serological indexes, and intracranial infection.

Variables	*r*	*P*
Cerebrospinal fluid test		
PCT, μg/L	0.669	.007
CRP, mg/L	0.701	.021
LA, nmol/L	0.568	.014
LDH, U/L	0.499	.009
Serological indicators		
AST, U/L	0.665	.048
LDH, U/L	0.714	.006
LDH-1, IU/L	0.622	.022

## 4 Discussion

In the process of patients with intracranial aneurysm undergoing craniotomy, the blood-brain barrier is vulnerable to severe damage, which greatly increases the risk of intracranial infection.^[13]^ Intracranial infection is one of the most serious clinical diseases of the central nervous system, and most common intracranial infections are bacterial infections.^[14,15]^ Intracranial infections are more complicated and difficult to treat.^[16]^ Long-term infections may affect normal brain nerve function and seriously affect the prognosis of patients. Timely and accurate prediction of intracranial infection is of positive significance for the clinical treatment of patients.^[17]^ We have found that the diabetes, intraoperative aneurysm rupture, intraoperative cerebrospinal fluid leakage, duration of surgery, and estimated blood loss were the independent risk factors of postoperative intracranial infection in patients with aneurysm surgery; early preventions are needed in clinical practice to reduce the postoperative intracranial infection.

Studies^[15,18]^ have shown that high blood sugar status in diabetic patients makes local capillaries a good medium for infection. After infection occurs, the level of inflammatory factors increases, resulting in a decrease in the patient's local immune function, and eventually intracranial injury is aggravated.^[19]^ Reports^[20–22]^ have shown that the recovery rate of intracranial infections in diabetic patients is significantly lower than in patients with normal blood sugar. During the operation, CSF leakage and aneurysm rupture will increase the patient's CSF and abnormal components in the brain tissue increase the patient's local inflammatory response and endothelial damage, and seriously affect the patient's prognosis.^[23,24]^ When patients have the above risk factors, they should be alert to the possibility of intracranial infection, and timely follow-up reports to determine whether they are complicated by intracranial infection, so as to detect and treat early. Some studies^[25,26]^ believe that the leakage of CSF, operation time, and operation blood loss of patients is correlated with intracranial infection, which is consistent with the findings of this study.

Craniocerebral surgery can cause oxidative stress in the brain and even the whole body, and oxidative stress can cause inflammatory reactions and abnormal immune levels.^[27,28]^ Therefore, the increase in inflammatory indexes caused by oxidative stress is closely related to the prognosis of patients with craniocerebral surgery.^[29]^ The comparison of the CSF inflammatory indexes of the 2 groups of patients showed that the CSF indexes PCT, CRP, LA, LDH of the patients in the intracranial infection group were higher than those of the non-intracranial infection, and CSF indicators are positively correlated with intracranial infection.^[30]^ After the craniotomy, the patient's brain tissue is in a high-incidence period of stress, indicating that the central nervous system is affected.^[31]^ Under the continuous and enhanced state of stress, the level of local inflammatory response in the brain continues to increase.^[32,33]^ Moreover, we have found that the serum LDH, LDH-1 levels of patients in the intracranial infection group are significantly higher than those in the non-intracranial infection group, suggesting that the levels of myocardial enzymes in the patients were significantly increased. Intracranial infection may cause damage to the central nervous system, which has a certain impact on cardiomyocytes, and increases the risk of cardiac function and structural damage to the heart.^[34,35]^ Combining the results of CSF bacterial culture for intracranial infection, we have found that the top 3 bacteria are *K. pneumoniae, E. coli*, and *S. epidermidis*. The bacteria are mainly Gram-negative bacteria, which is more consistent with previous related research results. Therefore, the clinical choice of antibiotics should be the third-generation cephalosporin to effectively control the infection.

Several limitations in this present study should be highlighted. First, limited by collected data, risk factors include hospital stay, GCS, stay on ICU, cardiovascular risk profile; these factors are only partially presented in our work, and there may be other risk factors of postoperative intracranial infection in patients with aneurysm surgery. Besides, about 45% included patients had not undergone drug sensitivity analysis in this study; therefore, the results of drug sensitivity could not be included for data analysis. Second, the sample size in this present study is small; it may underpower to detect the associated influencing factors. It is worth noting that due to the limited number of cases in this study, and the lack of multicenter comparisons and prospective research reports, the treatment and prevention of postoperative intracranial infections need further investigations in the future.

## 5 Conclusion

We have found that the patient's diabetes, intraoperative aneurysm rupture, intraoperative CSF leakage, duration of surgery ≥360 minutes, and estimated blood loss ≥125 mL are the independent risk factors of postoperative intracranial infection in patients with aneurysm surgery. Patients with internal infections combined with these risk factors should be given special attention to achieve early detection, prevention and treatment of intracranial infections, and the detection of CSF indicators and serological indicators of patients can help the diagnosis intracranial infections in patients with intracranial aneurysms. The occurrence of postoperative infection in patients with intracranial aneurysm in neurosurgery is the result of a combination of many factors. To prevent the occurrence of intracranial infection after surgery, it requires the joint efforts of doctors and nurses and the patient's family members to actively intervene in the factors that may cause infection.

## Author contributions

JF, YW designed research; XG, JF, YW conducted research; XG analyzed data; XG wrote the first draft of manuscript; JF, YW had primary responsibility for final content. All authors read and approved the final manuscript.

**Investigation:** Xiaohong Guo, Junkang Fang, Yi Wu.

**Methodology:** Yi Wu.

**Project administration:** Xiaohong Guo, Yi Wu.

**Software:** Xiaohong Guo.

**Supervision:** Yi Wu.

**Validation:** Xiaohong Guo, Junkang Fang, Yi Wu.

**Visualization:** Junkang Fang, Yi Wu.

**Writing – original draft:** Xiaohong Guo, Junkang Fang, Yi Wu.
